# High prevalence of human papillomavirus and European variants of HPV 16 infecting concomitantly to cervix and oral cavity in HIV positive women

**DOI:** 10.1371/journal.pone.0227900

**Published:** 2020-04-22

**Authors:** Milagros Pérez-Quintanilla, Rocío Méndez-Martínez, Salvador Vázquez-Vega, Raquel Espinosa-Romero, Rita Sotelo-Regil, María Delia Pérez-Montiel, Ubaldo Ramos-Alamillo, Teresita de Jesús Cabrera-López, Salim Abraham Barquet-Muñoz, Carlos Pérez-Plascencia, Alejandro García-Carrancá, David Cantú de León

**Affiliations:** 1 Subdirección de Investigación Clínica, Instituto Nacional de Cancerología México (INCan), Secretaría de Salud (SSA), Mexico City, Mexico; 2 Unidad de Investigación Biomédica en Cáncer, Subdirección de Investigación Básica, Instituto Nacional de Cancerología, SS, Mexico City, Mexico; 3 Doctorado en Biotecnología & Doctorado en Ciencias Biomédicas, Facultad de Ciencias Químico-Biológicas, Universidad Autónoma de Sinaloa, Culiacán, Mexico; 4 Unidad de Investigación en Epidemiología y Servicios de Salud (UIESS), Centro Médico Nacional Siglo XXI, Instituto Mexicano del Seguro Social (IMSS), Mexico City, Mexico; 5 Servicio de Citopatología, INCan, SSA, Mexico City, Mexico; 6 Servicio de Patología Quirúrgica, INCan, SSA, Mexico City, Mexico; 7 Servicio de Ginecología, Clínica Especializada Condesa (CEC) de los Servicios de Salud Pública del Distrito Federal, SSA, Mexico City, Mexico; 8 Servicio de Ginecología Oncológica, INCan, SSA, Mexico City, México; 9 Unidad de Genómica y Cáncer, Subdirección de Investigación Básica, INCan, SSA and Facultad de Estudios Superiores Iztacala, Universidad Nacional Autónoma de México, Mexico City, Mexico; 10 Instituto de Investigaciones Biomédicas, Univerisdad Nacional Autónoma de Mexico, Mexico City, Mexico; 11 Subdirección de Investigación Clínica, INCan, SSA, México City, Mexico; Universidad de Chile, CHILE

## Abstract

Human papillomavirus (HPV) is the most common sexually transmitted infection worldwide, with a high prevalence and high transmissibility. High-risk HPV (hrHPV) infection is the primary cause of cervical cancer. The HPV variants present in the uterine cervix and oral cavity of HIV+ women have not been described.

**Objective:**

Identify the prevalence of HPV infections in the uterine cervix and oral cavity and HPV16 variants in HIV+ women.

**Methods:**

A total of 174 HIV+ women attended an HIV+ specialized clinic in Mexico City. Cells were obtained from the oral cavity and cervix to extract DNA. Polymerase chain reaction (PCR) was used to amplify the HPV sequence with generic primers. We detected specific HPV types using the INNO-LiPA HPV Genotyping Extra II Kit (INNOGENETICS). The identification of variants was studied by sequencing the E6 gene with a Big Dye Terminator Kit and an Applied Biosystems 3500/3500xL genetic analyzer.

**Results:**

HPV infection was very high in the uterine cervix (168/174, 96.6%) and oral cavity (161/174, 92.5%). The prevalence of HPV concurrent infections in the cervix and oral cavity was 155/174 (89.1%). We found hrHPVs to be more prevalent than low-risk HPVs (lrHPVs) in the oral cavity (90.2% versus 45.4%) and that infections simultaneously affected the cervix (94.3% versus 36.2%) and oral cavity (85.1% versus 20.1%). Surprisingly, only European variants of HPV type 16 were found in the uterine cervix of women and the oral cavity of all tested samples (52 oral cavity samples and 52 uterine cervix samples).

**Conclusions:**

The high prevalence of HPV, multiple infections and presence of the EP350G intravariant in both anatomical regions are strongly related to the persistence of the virus, which is fundamental for the development of cancer. Therefore, it is very important to control and monitor this high-risk population as well as implement programs for the early detection of HPV and vaccination.

## Introduction

Human papillomavirus (HPV) is the most common sexually transmitted disease worldwide [1]. The prevalence of HPV infection in women worldwide is estimated to range from 2–44% [1]. It has been demonstrated that the majority of reproductive-age women who are sexually active have been exposed to one or more types of HPV in the genitals (approximately 50%) and oral cavity (30%) [2].

HPV types are classified into low-risk (lrHPV) or high-risk (hrHPV) based on their capability to induce malignant lesions and show a characteristic tropism for epithelia (i.e., the oral and cervical mucosal lining) [3,4].

Infection with hrHPV could be aggravated by immunosuppression, such as that observed in HIV patients [2]. Developing countries present the highest prevalence of HPV [5] and HIV [6] infections. HPV types found in the uterine cervix and oral cavity of HIV-positive patients show a different distribution of prevalent types compared with those found in HIV-negative patients [7,8].

These variants have been associated with the transmission of HPV within specific populations and differ in their biological characteristics and oncogenic potential in HIV+ women [9,10]. HPV intertype variants have been suggested to play a critical role in cervical carcinogenesis and are recognized as important markers for research on viral transmission and persistence [11,12].

It is controversial whether there is a direct relationship between HPV and HIV infections [13,14]. It is clear that the behavioral, immunologic, and genetic factors of the host participate in the complex interactions between HIV and HPV infections [15]. Sexual intercourse seems to be the most important route of transmission of genital and oral HPV infection [16]. Reproductive antecedents and sexual behavior may be used as indicators for HPV infection risk. Several studies have reported that early sexual initiation by both oral and vaginal intercourse and the number of sex partners are highly significant.

Tobacco smoking has also been deemed important, although there is no consensus between studies [13]. Among the immunological and related characteristics of HIV patients, we considered the number of CD4+ cells, HIV viral load and the time living with HIV infection [15].

The aim of this study was to determine the prevalence of several HPV types and HPV16 variants in the uterine cervix and oral cavity, as well as in concurrent infections in both anatomical sites, in HIV-positive women and relate these findings to the presence of certain risk factors.

## Materials and methods

### Samples

We studied 174 HIV+ women who attended an HIV specialized clinic in Mexico City. Patients over 18 years old presented for care between 1 February 2014 and 28 February 2015. Cervical and oral samples were obtained with a cytobrush and inserted into a collection tube containing PreservCyt.

The current study was conducted according to the Declaration of Helsinki revised by the 58th Assembly of the World Medical Association in Edinburgh, Scotland, in October 2000, established norms (Official Mexican Norm NOM010 SSA2 2011) and the Regulations of the General Health Law, in Art 16 and Law 820 “Law of Promotion, Protection and Defense of Human Rights in the Presence of AIDS”. The study protocol was approved and reviewed by the Committee of Investigation and Ethics in Investigation of the Mexico City-based INCan (CI/061/14). It was also approved by Mexico´s Federal Commission for the Protection of Health Risk (COFEPRIS) (143399419a151/2014) for the obtaining and processing of biological samples and for the transport of the samples from Nicaragua to Mexico. Written informed consent was obtained from all the participants.

### Data collection

Sociodemographic, clinical and sexual behavior information was collected using a self-applied written questionnaire with assistance from one of the researchers who aided participants if needed, having been previously assigned a confidentiality code.

### CD4 cell count and viral load

To determine the CD4 cell count and viral load, a peripheral blood sample was obtained at the time of HIV diagnosis and processed by flux photometry (for the CD4 cell count: CD4 cells/mm^3^) and amplified by polymerase chain reaction (PCR) (for viral load) in a time not exceeding 18 hours in the same laboratory. All the patients received antiretroviral therapy (ART), and the CD4 cell count and viral load were assessed to evaluate the adherence support of treatment with monitoring every 3–6 months. For this research, we considered the last CD4 cell count and viral load because all the women in the study received ART.

### Extraction of DNA

Next, 600 μl of nuclear lysis solution and 17.5 μl of proteinase K were added to the samples and incubated at 55°C for 15 hours. Subsequently, 1.5 μl of RNase was added and allowed to act for 30 minutes at 37°C. Then, 200 μl of precipitating protein solution was added, stirred to homogenize, incubated for 5 minutes on ice and centrifuged at 14,000 rpm for 5 minutes. The supernatant was transferred to a new tube, 500 μl of isopropanol was added, and the mixture was stirred manually and centrifuged for 1 minute at 14,000 rpm at room temperature. The supernatant was decanted, and the tubes were allowed to dry inverted. Finally, 50 μl of rehydrating reuptake solution was added by pipetting, and the DNAs were incubated for 1 hour at 65°C, quantified on an Epoch spectrophotometer and stored at -20°C.

### Detection of HPV

HPV was detected using GP5+ (TTTGTTACTGTGGTAGATACTAC) and GP6+ (GAAAAATAAACTGTAAATCATATTC) primers, which detect a highly conserved region of the L1 gene of different HPVs (150 bp). The negative samples were subjected to a second PCR with primers for GH20/PC04 that amplify a fragment of the β-globin gene (GH20 (GAAGAGCCAAGGACAGGTAC), PC04 (CAACTTCATCCACGTTCACC) (280 bp)), which allowed us to identify the integrity of the DNA and corroborate whether the samples were negative in the presence of HPV.

The PCR protocol (25 μl) was as follows: 1X buffer, 3 mM MgCl2, 0.2 mM dNTPs, 10 pmol/μl of each oligonucleotide primer, and 1.0 U Taq DNA polymerase (PROMEGA). The PCR conditions were as follows: 1 cycle at 94°C/10 min; 40 cycles (94°C/30 sec, 48°C/30 sec, 72°C/45 sec) and 1 cycle at 72°C/7 min. The products were analyzed by electrophoresis in 1.5% agarose gels and visualized with *Gel Red*.

### Viral types and genotyping

INNO-LiPA HPV Genotyping Extra II is based on the principle of reverse hybridization. A fragment of the L1 region of the human papillomavirus (HPV) genome is amplified using SPF10 Plus primers. This technology provides high test sensitivity due to the precision of the short 65-bp PCR product and permits the simultaneous detection of multiple genotypes in a single sample. The resulting biotinylated amplicons are then denatured and hybridized with specific oligonucleotide probes. An additional primer pair for the amplification of the human HLADPB1 gene is added to monitor sample quality and extraction and for conjugate control. All probes are immobilized as parallel lines on membrane strips. After hybridization and stringent washing, streptavidin-conjugated alkaline phosphatase is added, which binds to any biotinylated hybrid previously formed.

### Variant identification

HPV type 16+ samples were subjected to PCR using primers specific to the E6 gene. Samples were subjected to full-length sequence analysis of a 215 bp fragment of the E6 gene (GCAACAGTTACTGCGACGTG/GGACACAGTGGCTTTTGACA). The PCR protocol (25 μl) was as follows: 1X buffer, 3 mM MgCl2, 0.2 mM dNTPs, 10 pmol/μl of each oligonucleotide primer, and 1.0 U Taq DNA polymerase (PROMEGA). The PCR conditions were as follows: 1 cycle at 95°C/7 min; 40 cycles (95°C/35 sec, 60°C/45 sec, 72°C/45 sec) and 1 cycle at 72°C/5 min.

The PCR products were sequenced using the BigDye Terminator Kit and an Applied Biosystems 3500/3500xL genetic analyzer. The sequence analysis was performed with the Chromas Program, and multiple sequence alignment was performed with Clustalw.

### Statistical analysis

Quantitative variables with a normal distribution are expressed as the mean and standard deviation (SD). Variables with a nonnormal distribution are expressed as the median and interquartile range (IQR). The prevalence of HPV is reported as the proportion of cervical, oral and cervical-oral samples positive for each identified viral type. The bivariate analysis was performed by *X*^*2*^ (Pearson). A multivariate analysis (bivariate logistic regression) was also performed to obtain the adjusted odds ratio (aOR). The analysis was performed for each type of HPV detected, for each group (lrHPV, hrHPV, and HPV16-18; analyzed as a group because all contribute to 70% of all cancer cases), and for “other hrHPV”, which excluded types 16 and 18. Variables that showed statistical significance for each logistic regression model are represented in tables. SPSS ver. 25 statistical software (IBM Corp.) was used.

## Results and discussion

### Sociodemographic characteristics

The average age of women in this study was 40.6 years (± 12.52). The median age at which subjects first had vaginal sex was 17 years (IQR: 15–19 years). The median age at which subjects first had oral sex intercourse was 16 years (IQR: 16–20 years). The median number of vaginal sex partners was 3 (IQR: 2–5), and the median number of oral sex partners was 2 (IQR: 1–3). Regarding smoking habits, 35% of the patients reported smoking between 1 and 40 cigarettes daily. Sixty-five percent of the patients did not smoke. Regarding HIV, the median time living with HIV infection reported by the patients was ≥5 years (IQR: 1–11 years), varying between 0 and 28 years. The median number of CD4 cells/mm^3^ of blood was 408 (IQR: 208–602 CD4 cells/mm^3^), varying between 7 and 1,800 CD4 cells/mm^3^. Most patients (56.3%) had an undetectable viral load (≤50 copies of HIV/mm^3^), and 43.7% of patients had a detectable number of HIV copies (Table 1).

**Table 1:** Sociodemographic, clinical, and sexual behavior baseline data of HIV-positive women included in this study. Number of patients = 174.

Characteristic	MCT or F	DM or (%)
Age (years)	40.6	±12.52
Age at first vaginal intercourse (years)	17	(15, 19)
Number of vaginal sex partners	3	(2, 5)
Age at first oral intercourse (years)	16	(16, 20)
Number of oral sex partners	2	(1, 3)
Smoking status		
No	113	65%
Yes	61	35%
Laboratory data		
Viral load (HIV copies/mm^3^)		
Undetectable (≤40–50 copies/mm^3^)	98	56.3%
Detectable (>51 copies/mm^3^)	76	43.7%
CD4 (cells/mm^3^)	408	(208, 602)
Time lapse after HIV diagnosis (years)	5	(1, 11)

Data are shown as the mean, standard deviation (±). Continuous variables are shown as the median and interquartile range. Categorical variables are shown as the number of patients and percentage. MCT = Measure of central tendency (mean or median), F = Frequency, DM = Dispersion measure (standard deviation, ±) or (%) = percentage.

### Prevalence of HPV types in oral cavity, uterine cervix and concurrent cervical-oral infections

**Fig 1:**
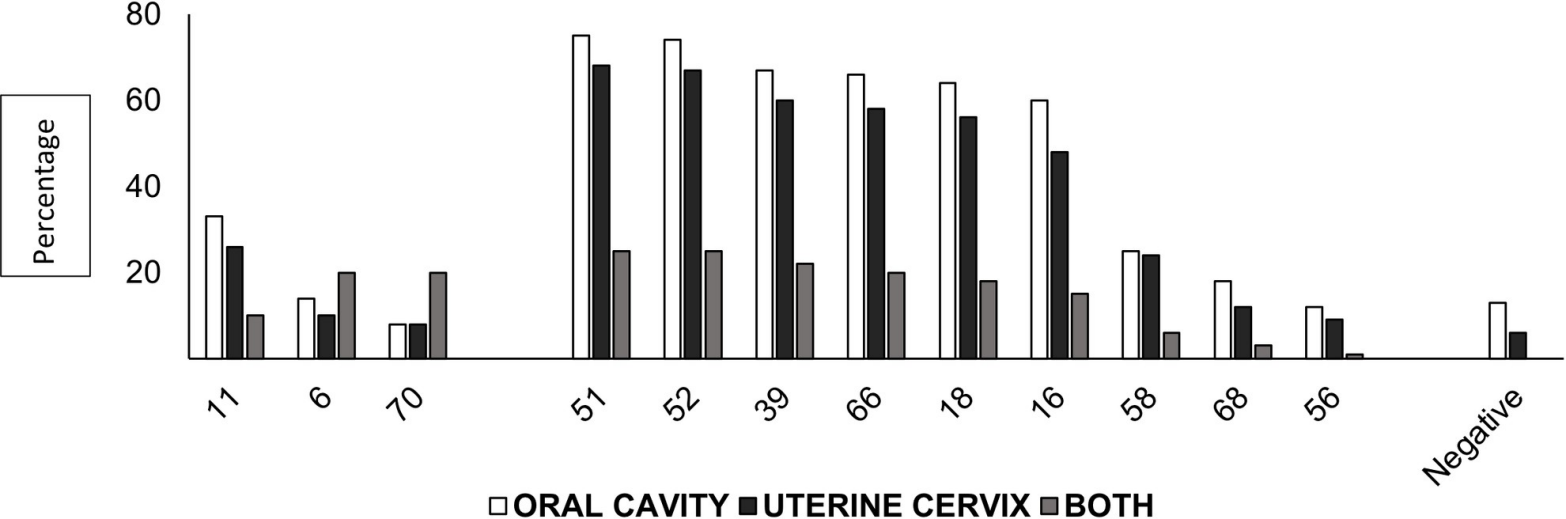
Summary of HPV prevalence in different anatomical sites. The prevalence of high-risk viral types is indicated according to their localization in the anatomical region (oral cavity, uterine cervix and both anatomical regions).

Using the INNO-LiPA HPV Genotyping Extra II Kit, which identifies 32 HPV types, we confirmed infections with 12 viral types (lrHPV types were 6, 11 and 70 and hrHPV types 16, 18, 39, 51, 52, 56, 58, 66 and 68). The prevalence of infection by all types of HPV in the cervix was 96.6%; that in the oral cavity was 92.5%; and that in both anatomical sites was 89.1%. The prevalence of lrHPV infections was lower than that of hrHPV infections in the oral cavity, cervix and both locations. The prevalence of each hrHPV type was slightly lower in the uterine cervix than in the oral cavity, and the patients who presented the same high-risk viral type in both regions had the highest percentage, corresponding to 25% (Fig 1).

**Fig 2:**
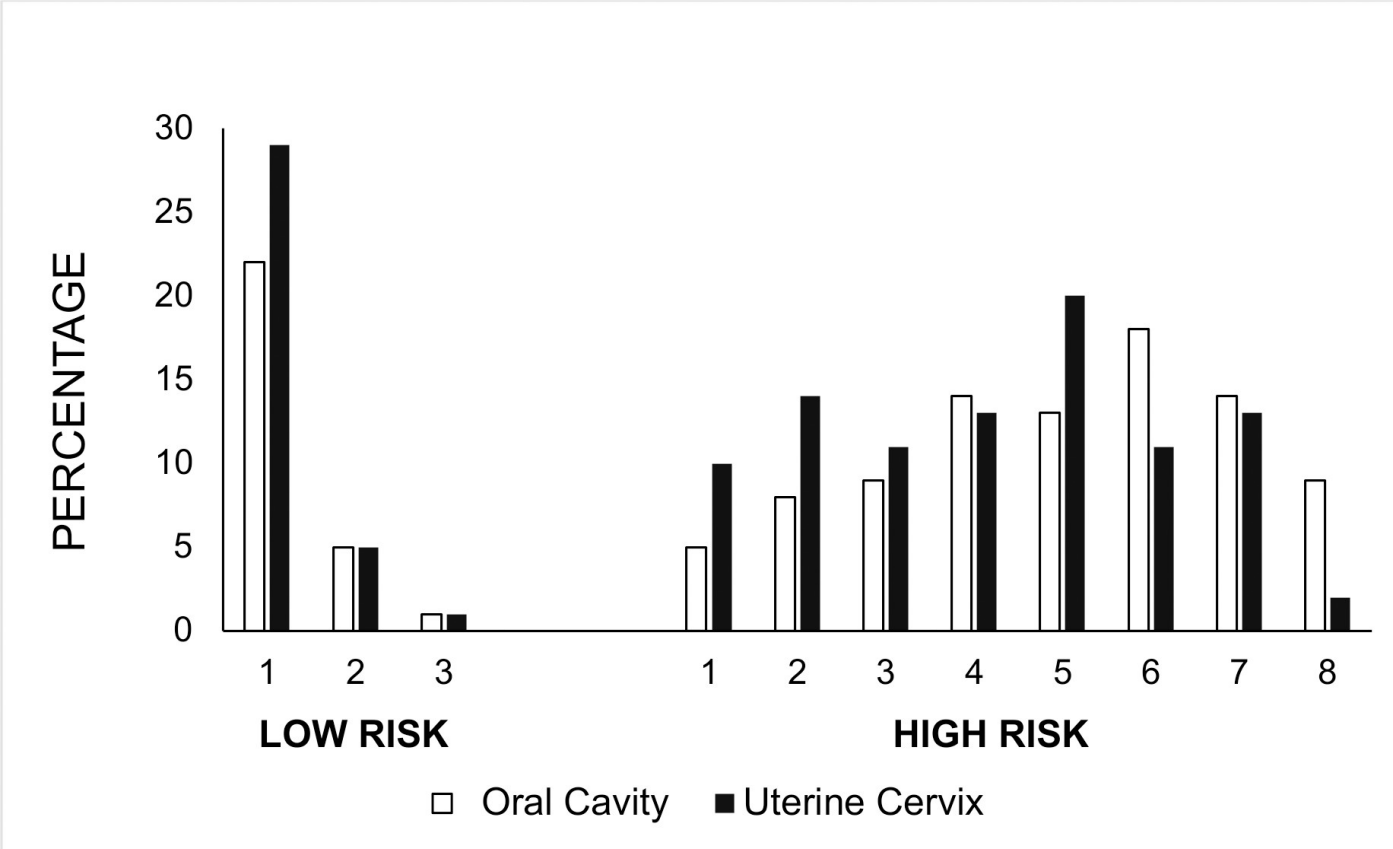
Graphical representation of the percentage of viral types present in patients. The number of types detected in the uterine cervix (black bars) and oral cavity (white bars) is shown.

The prevalence of HR viral types in the **oral cavity (OC) and uterine cervix (UC)** was as follows: 5% OC and 10% UC for 1 viral type, 8% OC and 14% UC for 2 viral types, 9% OC and 11% UC for 3 viral types, 14% OC and 13% UC for 4 viral types, 13% OC and 20% UC for 5 viral types, 18% OC and 11% UC for 6 viral types, 14% OC and 13% UC for 7 viral types and 9% OC and 2% UC for 8 viral types. For the low-risk types, a difference was found only in the presence of 1 viral type (22% in the oral cavity and 29% in uterine cervix); for the cases of 2 and 3 viral types, the percentages were the same (5% and 1%, respectively) (Fig 2).

### Risk factors for and probability of HPV infection

#### Oral cavity

The risk of HPV16 infection in the oral cavity was significantly greater for women with 2 and 3 or more oral sex partners (aOR = 4.58, CI_95%_ = 1.67–12.57 and aOR = 8.04, CI_95%_ = 2.49–25.95, respectively). Additionally, ≤ 200 CD4 cells/mm^3^ was associated with a significantly increased risk of infection (aOR = 2.73, CI_95%_ = 1.06–7.0). Moreover, HPV18 risk infection was important for those living with HIV for ≥ 5 years (aOR = 2.62, CI_95%_ = 1.18–5.8). The risk of simultaneous infection with HPV16 and HPV18 was greater in patients with 2 and 3 or more oral sex partners (aOR = 3.41, CI_95%_ = 1.25–9.27 and aOR = 4.36, CI_95%_ = 1.42–13.4, respectively). Regarding CD4 cells/mm^3^, a count ≤ 200 was also associated with a significant risk of HPV16 infection (aOR = 2.57, CI_95%_ = 1.04–6.09). The risk of infection with HPV66 in the same region was significant in patients reporting a start of oral sexual activity at ≤16 years of age (aOR = 2.92, CI_95%_ = 1.20–7.05), as well as in patients with 2 and 3 or more oral sex partners (aOR = 3.20, CI_95%_ = 1.16–8.79 and aOR = 8.45, CI_95%_ = 2.48–28.7, respectively) and in those living with HIV for ≥ 5 years (aOR = 3.28, CI_95%_ = 1.37–7.86). The risk for HPV68, hrHPV (as a group), and HPV51 oral infection was greater in patients who reporting smoking (aOR = 5.89, CI_95%_ = 2.34–14.86, aOR = 9.13, CI_95%_ = 1.09–75.89 and aOR = 3.62, CI_95%_ = 1.54–8.74, respectively). For HPV39, the risk of oral infection was important only in women living with HIV for ≥ 5 years (aOR = 2.48, CI_95%_ = 1.12–5.46) (Table 2).

**Table 2:** Risk of hrHPV infections in the oral cavity associated with sociodemographic and clinical factors (OR = adjusted odds ratio calculated by binary logistic regression; CI_95%_ = confidence interval at 95%).

ORAL CAVITY									
		HPV16	HPV18	HPV16-18	HPV66	HPV68	hrHPV	HPV51	HPV39
		aOR (CI95%)							
**Risk factor**	**Total n (%)**								
**Smoking**									
**No**	**113 (65.0)**					**Ref.**	**Ref.**	**Ref.**	
**Yes**	**61 (35.0)**					**5.89 (2.34–14.86)**	**9.13 (1.09–75.89)**	**3.62 (1.54–8.74)**	
**Age at first oral intercourse**									
**≥17**	**69 (39.7)**				**Ref.**				
**≤16**	**105 (60.3)**				**2.92 (1.20–7.05)**				
**Number of oral sex partners**									
**1**	**30 (17.2)**	**Ref.**		**Ref.**	**Ref.**				
**2**	**59 (33.9)**	**4.58 (1.67–12.57)**		**3.41 (1.25–9.27)**	**3.20 (1.16–8.79)**				
**≥3**	**44 (25.3)**	**8.04 (2.49–25.95)**		**4.36 (1.42–13.4)**	**8.45 (2.48–28.7)**				
**Without a response**	**41 (23.6)**								
**CD4 (cells/mm3)**									
**>500**	**68 (39.1)**	**Ref.**		**Ref.**					
**201–499**	**64 (36.8)**								
**≤ 200**	**42 (24.1)**	**2.73 (1.06–7.0)**		**2.57 (1.04–6.09)**					
**Time lapse after HIV diagnosis (years)**									
**≤ 4**	**82 (47.1)**		**Ref.**		**Ref.**				**Ref.**
**≥ 5**	**92 (52.9)**		**2.62 (1.18–5.8)**		**3.28 (1.37–7.86)**				**2.48 (1.12–5.46)**

Only significant values are presented. Ref. = Reference category.

#### Uterine cervix

HPV16 risk infection was greater with a viral load of ≥ 51 copies of HIV/mm^3^ (aOR = 2.49, CI_95%_ = 1.26–4.88). HPV18 risk infection was important in those living with HIV for ≥ 5 years (aOR = 2.24, CI_95%_ = 1.04–4.8). The finding of ≥ 51 copies of HIV/mm^3^ was associated with a significant risk (aOR = 2.76 (CI_95%_ = 1.36–5.52) of simultaneous HPV16-18 infection. The risk of HPV68 infection in the uterine cervix was significant in women living with HIV for ≥ 5 years (aOR = 7.47, CI_95%_ = 1.7–32.6). Regarding lrHPV (as a group), risk infection was significant only in women ≥ 45 years old (aOR = 2.64, CI_95%_ = 1.1–6.84) and with a detectable HIV viral load (aOR = 2.18, CI_95%_ = 1.1–4.32) (Table 3).

**Table 3:** Risk of hrHPV infections in the uterine cervix associated with sociodemographic and clinical factors (OR = adjusted odds ratio calculated by binary logistic regression; CI_95%_ = confidence interval at 95%).

UTERINE CERVIX						
		HPV16	HPV18	HPV16-18	HPV68	lrHPV
		aOR (CI95%)				
**Risk factor**	**Total n (%)**					
**Age**						
**≤33**	**56 (32.2)**					**Ref.**
**34–44**	**58 (33.3)**					
**≥45**	**60 (34.5)**					**2.64 (1.1–6.84)**
**Viral load (copies HIV/mm3)**						
**Undetectable (≤50 copies/mm3)**	**98 (56.3)**	**Ref.**		**Ref.**		**Ref.**
**Detectable (≥51 copies/mm3)**	**76 (43.7)**	**2.49 (1.26–4.88)**		**2.76 (1.36–5.52)**		**2.18 (1.1–4.32)**
**Time lapse after HIV diagnosis (years)**						
**≤ 4**	**82 (47.1)**		**Ref.**		**Ref.**	
**≥ 5**	**92 (52.9)**		**2.24 (1.04–4.8)**		**7.47 (1.7–32.6)**	

Only significant values are presented. Ref. = Reference category.

#### Oral cavity-uterine cervix

The risk of concurrent cervical-oral infection by HPV18 was associated only with living with HIV for ≥ 5 years (aOR = 2.73, CI_95%_ = 1.14–6.50). The risk of concurrent cervical-oral infection by HPV16-18 was greater in patients with 3 or more oral sex partners (aOR = 5.95, CI_95%_ = 1.28–27.66) and in those living with HIV for ≥ 5 years (aOR = 4.33, CI_95%_ = 1.54–12.13). The risk of HPV66 infection in both anatomical regions was high only in patients reporting an onset of oral sexual activity at ≤16 years of age (aOR = 3.48, CI_95%_ = 1.49–8.13) and in those living with HIV for ≥ 5 years (aOR = 66.5, CI_95%_ = 2.7–160.5). Regarding concurrent cervical-oral HPV68 infection, smoking increased the risk (aOR = 25.0, CI_95%_ = 2.26–276.2). Finally, the risk of concurrent cervical-oral infection by HPV39 was significant only in women living with HPV for ≥ 5 years (aOR = 2.29, CI_95%_ = 1.1–5.17) (Table 4).

**Table 4:** Risk of concurrent cervical-oral hrHPV infections associated with sociodemographic and clinical factors (OR = adjusted odds ratio calculated by binary logistic regression; CI_95%_ = confidence interval at 95%).

CONCURRENT ORAL CAVITY & UTERINE CERVIX						
		HPV18	HPV16-18	HPV66	HPV68	HPV39
		aOR (CI95%)				
**Risk factor**	**Total n (%)**					
**Smoking**						
**No**	**113 (65.0)**				**Ref.**	
**Yes**	**61 (35.0)**				**25.0 (2.26–276.2)**	
**Age at first oral intercourse**						
**≥17**	**69 (39.7)**			**Ref.**		
**≤16**	**105 (60.3)**			**3.48 (1.49–8.13)**		
**Number of oral sex partners**						
**1**	**30 (17.2)**		**Ref.**			
**2**	**59 (33.9)**					
**≥3**	**44 (25.3)**		**5.95 (1.28–27.66)**			
**Without a response**	**41 (23.6)**					
**Time lapse after HIV diagnosis (years)**						
**≤ 4**	**82 (47.1)**	**Ref.**	**Ref.**	**Ref.**		**Ref.**
**≥5**	**92 (52.9)**	**2.73 (1.14–6.50)**	**4.32 (1.54–12.1)**	**66.5 (2.7–160.5)**		**2.29 (1.1–5.17)**

Only significant values are presented. Ref. = Reference category.

#### HPV16 E6 gene variants

**Fig 3:**
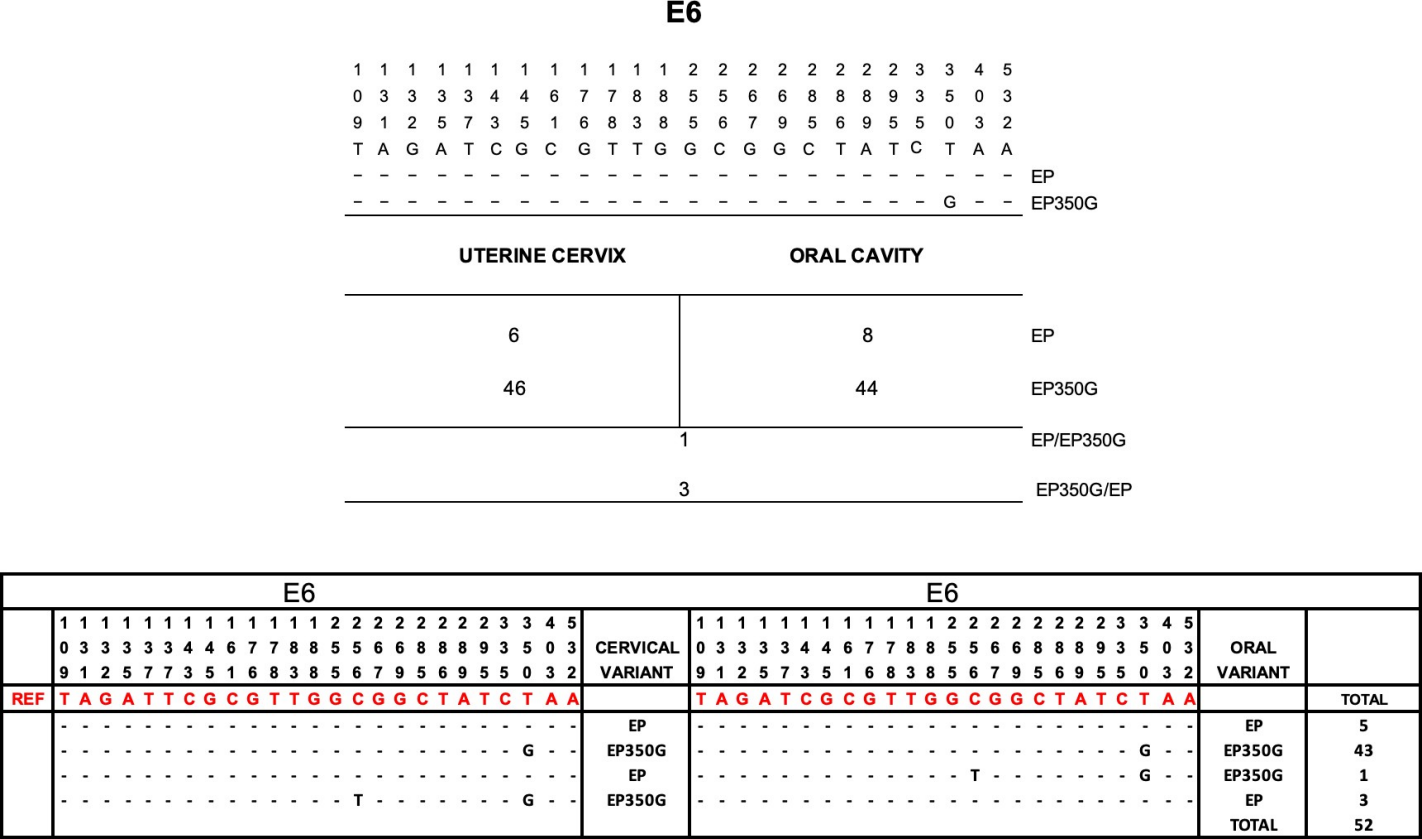
E6 region sequences representative of HPV16 variants. Summary of the variants identified in the oral cavity and uterine cervix. The predominant variant in both anatomical regions was the European variant, an intravariant EP 350G.

We analyzed 104 samples from 52 patients (52 oral cavity samples and 52 uterine cervix samples) to identify E6 gene variants. The European variant is predominant in the oral cavity and uterine cervix. The prevalence of intravariants was the European 350G (EP350G) variant (86.5%, 90 samples), followed by the European prototype (EP) variant (24.13%, 14 samples). In total, 80.77% (n = 42) of patients harbored EP350G in both anatomical regions, and 9.6% (n = 5) of patients harbored EP in both regions. Conversely, 5.76% (n = 3) of patients harbored the EP variant in the oral cavity and EP 350G in the uterine cervix. Only one patient harbored EP350G in the oral cavity and EP in the uterine cervix (Fig 3).

This cross-sectional study on women from Mexico City living with HIV determined the prevalence of HPV types, HPV16 E6 variants and risk factors associated with infection in the uterine cervix, oral cavity, concurrent cervical-oral infection.

The prevalence of infection by any of the 12 HPV types detected in this study in the uterine cervix was 96.6%. It has been reported that the prevalence of HPV infection with any of the 37 HPV types of low and high oncogenic risk in HIV+ women who receive antiretroviral treatment is also high (83%) [17]. The prevalence in our study is consistent with the history that HPV infections are more prevalent and persistent in women infected with HIV [18].

It has been suggested that the prevalence of HPV in the oral cavity in HIV+ women is considerably low [19]. In our study, the prevalence of infection with any of the 12 HPV types considered was 92.5%, which contrasts with the range of 5.6%-34% in women living with HIV [12,20]. Other studies in HIV+ individuals have reported an HPV prevalence in the oral cavity ranging from 1.2% to 87%, which depends on the population studied and the method of detection chosen. [21] [22] Our data suggest that oral HPV infection varies widely in HIV+ individuals [23].

It has also been reported that an increase in oral HPV infection, mainly with concurrent genital HPV infection, follows a positive trend over time [24]. According to our data, obtained from women approximately 40.6 years of age (± 12.52), the prevalence of concurrent infection of any of the 12 types of HPV in the uterine cervix and oral cavity was 89.1%. It has been described that women with concurrent infection with HPV in the uterine cervix and oral cavity may have a high susceptibility to infection by this virus, either due to hereditary or immunological vulnerability. Another explanation is the change in sexual behavior that increases the risk of oral and cervical HPV infection [25].

Regarding infection by low or high oncogenic risk types, in our study, the prevalence of lrHPV was lower than the prevalence of hrHPV in the uterine cervix and oral cavity, similar to that reported in Brazilian women with HIV. The prevalence of each of the 9 types of hrHPV identified in this study was slightly lower in the uterine cervix than in the oral cavity. It has been described that the oral cavity could be a reservoir of HPV infection, which probably explains this finding [26].

We found the same hrHPV in the uterine cervix and oral cavity. The frequency of the viral types described in HIV-positive individuals is different from that described in HIV-negative individuals, where HPV16 is the most prevalent. This change indicates that immunosuppression is involved. On the other hand, most of the hrHPV infections were multiple, and in the uterine cervix, 20% of the samples had 5 viral types, while in the oral cavity, 18% of the samples had 6 viral types [18].

The prevalence of infection by the same type of hrHPV in the uterine cervix and oral cavity was not greater than 25%. A meta-analysis of the concomitant prevalence in the cervix and oral cavity in HIV-positive and HIV-negative women showed a prevalence of 46.8% and 15.6%, respectively. In women positive for HIV, immunity explains these values [27]. The clinical and epidemiological importance of this concordance in both anatomical sites lies in the factors associated with the infection, involved in simultaneous transmission, persistence and/or viral autoinoculation [25,28].

The presence of HPV is variable according to the study population. However, it has been reported that women with genital HPV are at an increased risk of having oral HPV [26]. We observed this correlation between HPV found in the cervix and in the oral cavity. We found that the most prevalent types were 51, 52, 66 and 16.

In this study, we present the risk factors for infection in the uterine cervix, oral cavity and concurrent locations (uterine cervix-oral cavity) both individually and as a group: lrHPVs (including HPV6, 11 and 70) and hrHPVs (including HPV16, 18, 39, 51, 52, 56, 58, 66 and 68). The exposed risk factors are similar to those proposed in other studies, and the particularities of their risk change with respect to the type of HPV, as reported in a previous study [27].

It has been widely reported that the geographic region and the polymorphisms present determine variants that are associated with the development of cancerous lesions. Adolescent women infected with HIV and Brazilian and Italian women show a high prevalence of hrHPV infection, presenting as the predominant variant European 350G; however, the presence of the African variant suggests different sexual mixing behaviors [10,15, 27]. We found that HIV-positive women of Mexican origin harbor the European variant. It has been reported that non-European variants are more oncogenic [28]; however, we found a significant prevalence of the HPV16 variant EP350G, which is strongly associated with the development of cervical cancer.

The association of the HPV16 E6 variant with the risk of cervical cancer can be explained by good viral capacity (the ability to evade the host's immune system and establish a persistent infection) and/or an increase in carcinogenicity. Studies have suggested that the presence of the T350G change in the E6 gene of HPV16 may have biological advantages [29] that promote cell immortalization and downregulate E-cadherin [30], as well as improve MAPK signaling mediated by E6 and cooperative transformation with the deregulated Notch1 pathway [31].

The 350T-G substitution (E-350G) in the E6 gene of HPV16 has been proposed as an additional risk factor for persistent infection and the cytologic progression of grade 2/3 CIN and squamous cell carcinoma (SCC) [32,33]. This change leads to a substitution at residue 83 of the E6 protein of a leucine for a valine (L83V) [34]. Thus, the presence of E-350G is associated with good evasion capacity of the host immune system and/or an increase in oncogenic potential associated with the intrinsic biological properties of viral proteins [31].

Interestingly, HIV+ women who participated in this study exhibited a high percentage of coinfections with HPV16 in both anatomical regions and, most importantly, harbored only European variants of this viral type, in particular the prototype variant, suggesting a particular tropism or behavior of the variants of this viral type.

Our study had a number of limitations. We did not have an HIV-uninfected cohort of women for comparison. Additionally, we did not have data on the previous immunological status of HIV-infected women. Furthermore, we did not perform a long follow-up for the evaluated incidence and clearance rates in the oral cavity or in the cervix. The strength of our research is that it was performed on epidemiologic data from HIV-infected women with oral and cervical HPV.

## Conclusions

The high prevalence of HPV, multiple infections and presence of the EP350G intravariant in both anatomical regions demonstrate that the infection present in patients is derived from the same virus. The intravariant EP350G is closely related to the persistence of the virus in individuals and is fundamental for the development of HPV-related cancer. Therefore, it is very important to control and follow-up this high-risk population as well as to implement programs for the early detection of HPV and vaccination.
